# Early emergency readmission frequency as an indicator of short-, medium- and long-term mortality post-discharge from hospital

**DOI:** 10.1007/s11739-020-02599-3

**Published:** 2020-12-26

**Authors:** David Fluck, Paul Murray, Jonathan Robin, Christopher Henry Fry, Thang Sieu Han

**Affiliations:** 1 Department of Cardiology, Ashford and St Peter’s Hospitals NHS Foundation Trust, Guildford Road, Chertsey, Surrey, KT16 0PZ UK; 2 Department of Respiratory Medicine, Ashford and St Peter’s Hospitals NHS Foundation Trust, Guildford Road, Chertsey, Surrey, KT16 0PZ UK; 3 Acute Medical Unit, Ashford and St Peter’s Hospitals NHS Foundation Trust, Guildford Road, Chertsey, Surrey, KT16 0PZ UK; 4 School of Physiology, Pharmacology and Neuroscience, University of Bristol, Bristol, BS8 1TD UK; 5 Department of Endocrinology, Ashford and St Peter’s Hospitals NHS Foundation Trust, Guildford Road, Chertsey, Surrey, KT16 0PZ UK; 6 Institute of Cardiovascular Research, Royal Holloway, University of London, Egham, Surrey, TW20 0EX UK

**Keywords:** Health economics, Healthcare services, Readmission prevention, Quality of care

## Abstract

Frequent emergency readmissions, an indicator of quality of care, has been rising in England but the underlying reasons remain unclear. We examined the association of early readmissions with subsequent mortality in adults, taking into account the underlying presenting diagnoses and hospital length of stay (LOS). Data of alive-discharge episodes were prospectively collected between 01/04/2017 and 31/03/2019 in an National Health Service hospital, comprising 32,270 patients (46.1% men) aged 18–107 years (mean = 64.0, ± SD = 20.5 years). The associations of readmission frequency within 28 days of discharge and mortality within 30 days and 6 months of hospital discharge, and over a 2-year period were evaluated, adjusted for presenting diagnoses, LOS, age and sex during the first admission. Analysis of all patients 18–107 years (reference: no readmission) showed mortality within 30 days was increased for 1 readmission: event rate = 9.2%, odds ratio (OR) = 3.4 (95% confidence interval (CI) = 2.9–4.0), and ≥ 2 readmissions: event rate = 10.0%, OR = 2.6 (95%CI = 2.0–3.3), and within 6 months for 1 readmission: event rate = 19.6%, OR = 3.0 (95%CI = 2.7–3.4), and ≥ 2 readmissions: event rate = 27.4%, OR = 3.4 (95%CI = 2.9–4.0), and over a 2-year period for 1 readmission: event rate = 25.5%, hazard ratio = 2.2 (95%CI = 2.0–2.4), and ≥ 2 readmissions: event rate = 36.1%, hazard ratio = 2.5 (95%CI = 2.2–2.8). Within the age groups 18–49, 50–59, 60–69, 70–79 and ≥ 80 years, readmissions were also associated with increased risk of mortality within 3 months and 6 months of discharge, and over 2-year period. In conclusion, early hospital readmission predicts short-, medium- and long-term mortality post-discharge from hospital in adults aged 18–107 years, independent of underlying presenting conditions, LOS, age and sex. Further research focussing on safe discharge and follow-up patient care may help reduce preventable readmissions and post-discharge mortality.

## Introduction

Emergency readmission frequency has been a major focus of research of healthcare services, used as a measure of quality of care and cost-efficiency, primarily in older adults [1–4]. A study of over 14 million emergency admissions in US adults ≥ 18 years revealed that the cost of a readmission within 30 days of discharge from hospital was about US $10,000 for sepsis and US $8500–9500 for respiratory and cardiac conditions [2]. Another US study showed the cost of an early readmission after a discharge for coronary artery bypass graft was greater—$13,500 [4]. Early emergency readmissions are continuously recorded by National Health Service (NHS) hospitals. Healthwatch England analysed data from 70 out of 125 hospitals and found that in 2017–18, the number of emergency readmissions increased by 9%; the fastest rate over the previous 5 years. There were 484,609 emergency readmissions to hospital within 30 days, a 22% rise over the previous 5 years, with numbers readmitted within 24 h of discharge (‘failed discharge’) from hospital rose by 33% in the same period [5, 6]. The underlying reasons for these increases in readmissions remain unclear and has led Healthwatch England to conclude that “Most troubling is that the sector still cannot report on how many emergency readmissions were unavoidable and which ones could be prevented, or use this insight to learn” [5]. However, increased readmission could be explained, in part, by the rising admissions in recent years. The total annual number of hospital admissions across all English hospitals in 2006 was 11 million, which rose by 28% to over 14 million in 2016. The corresponding figures for those older than 65 years were 4 million in 2006 and 6 million in 2016, a rise of 46%, [7] while the trends in hospital length of stay (LOS) for those staying at least one night showed a marginal increase between 2010 and 2014, from 6.85 to 6.92 days [8].

Other than being an indicator of quality of care, early emergency readmission also reflects poor health status of an individual [9–11]. Hitherto, there is a paucity of data on its relationship with health consequences, such as mortality. In this study, we examined the associations of early readmission frequency on all-cause mortality within 30 days and 6 months after discharge from hospital and over a 2-year period, in adults aged 18–107 years, taking into account the presenting diagnoses and LOS.

## Methods

### Participants and setting

In line with the NHS, information for every patient attending emergency department is recorded using Patient Administration System (PAS) including demographics, diagnosis, co-morbidities, dates of admission and discharge. Data of consecutive alive-discharge episodes over 2 years between 1 April 2017 and 31 March 2019 extracted from PAS in a single NHS hospital [12, 13].

### Outcome measures

Information on the frequency of unplanned admissions and all-cause readmissions within 28 days, and all-cause mortality within 30 days and 6 months after hospital discharge, and over 2-year period was recorded. Primary diagnoses presented in the first admission were coded according to international classification of diseases (ICD-10) [14]. Cancer and obstetrics spells were excluded in line with the NHS data collection for general hospital admissions [15]. LOS was calculated as the duration between the dates of first admission and first discharge.

### Categorisation of variables

Age was categorised by decades from 50 years old: 50–59, 60–69 and 70–79 years. All those aged 18–49 years were grouped together due to low mortality rates, while those aged 80–107 years were combined together due to small numbers. The frequency of readmissions within 28 days of discharge was categorised into three groups: No readmission, readmitted once, and readmitted ≥ 2 times. Prolonged LOS was considered as those staying in hospital ≥ 2 weeks (top 10th centile of LOS).

### Statistical analysis

Continuous data are presented as mean ± standard deviation, except some skewed data sets that are shown as median values (25%, 75% interquartiles). Chi-square tests were used to assess the relationship between readmission frequency and mortality, and Kruskal–Wallis *H* test to assess differences in LOS between different categories of readmission frequency. The frequency of readmission was used to predict mortality within 30 days and within 6 months of hospital discharge using multivariable stepwise logistic regression, and mortality over a 2-year period using multivariable stepwise Cox regression. Kaplan–Meier curves were constructed to examine survival time after hospital discharge in relation to readmission frequency. Data were adjusted for age and sex and primary diagnosis at presentation and prolonged LOS during the first admission for all ages and for different age categories. Odds ratio (OR) and hazard ratio (HR) are given with 95% confidence intervals (CI). Analyses were performed using IBM SPSS Statistics, v25.0 (IBM Corp., Armonk, NY).

## Results

### Subject characteristics

A total of 14 878 men and 17 392 women of mean age 64 ± 20.5 years were studied. Table 1 shows patient characteristics including primary diagnoses presented in the first admission and age distribution. The proportions of patients with no readmission, one readmission and ≥ 2 readmissions within 28 days of first hospital discharge were 88.5, 8.1 and 3.3%, and over a 2-year period were 78.4, 13.5 and 8.1%, respectively. Readmission rates increased incrementally with age groups 18–49, 50–59, 60–69, 70–79 and ≥ 80 years from 5.0, 5.8, 6.7, 8.0, to 13.7% for one readmission and 1.0, 1.5, 2.4, 3.3 to 6.7% for ≥ 2 readmissions, respectively. There were 2.6, 6.8, and 10.2% of patients who died within 30 days, after 6 months and after 2 years post discharge, respectively. The mean age of death (81 ± 12 years) was similar among all groups.

**Table 1:** Characteristics of 14,878 men aged 18.0–104.1 years (mean = 63.9 ± SD = 19.3 years) and 17,392 women aged 18.0–106.7 (mean = 64.1 ± SD = 21.6 years)

	*n*	%
Age distribution		
18–49.9 years	8403	26.0
50–59.9 years	4304	13.3
60–69.9 years	4739	14.7
70–79.9 years	6068	18.8
≥ 80 years	8756	27.1
Primary diagnosis on first admission		
Sepsis	1170	3.6
Viral infections	145	0.4
Haematological disorders	676	2.1
Metabolic and endocrine disorders	1084	3.4
Diabetes^b^	350	1.1
Psychiatric disorders	279	0.9
Neurological disorders	722	2.2
Ophthalmic disorders	163	0.5
Cardiovascular disorders	4416	13.7
Congestive heart failure^b^	405	1.3
Pulmonary disorders	3367	10.4
Asthma^b^	207	0.6
Chronic obstructive pulmonary disease^b^	470	1.5
Pneumonia^b^	1692	5.2
Gastrointestinal disorders	4144	12.8
Dermatological disorders	1083	3.4
Musculoskeletal disorders	3169	9.8
Urological disorders	3686	11.4
Urinary tract infection^b^	1116	3.5
Bodily pain	4344	13.5
Bone fractures	2624	8.1
Hip fractures^b^	639	2.0
Medical device-related complications	1015	3.1
Others (not specified)	183	0.6
Stayed in hospital ≥ 2 weeks	3228	10.0
Number of readmissions within 28 days of discharge		
None	28,548	88.5
Once	2666	8.3
≥ 2 times	1056	3.3
Mortality status		
Death within 30 days of first admission	834	2.6
Death within 6 months of first admission^a^	2192	6.8
Death within a 2-year period	3305	10.2

^a^This group includes those who died within 30 days of admission

^b^Specific conditions within categories of disorders

### Association of primary diagnoses and LOS during admission with readmission frequency

Admissions for sepsis, diabetes, psychiatric, congestive heart failure, chronic obstructive pulmonary disease, pneumonia, urinary tract infection, musculoskeletal disorders and bodily pain, medical device-related complications, and prolonged LOS all linked to subsequent readmissions within 28 days of charge from hospital (Table 2). The median (interquartile range) of the preceding hospital LOS during the first hospital admission was 2.1 (0.9–5.1) days for no readmission, 6.6 (2.7–13.3) days for 1 readmission and 11.3 (6.7–19.8) days for ≥ 2 readmission (Kruskal–Wallis *H* test for group differences: *χ*^2^ = 1765, *p* < 0.001).

**Table 2:** Health conditions presented as primary diagnoses and prolonged length of stay during first hospital admission in relation to subsequent readmission frequency

Primary diagnosis on first admission	Disorders from first admission in relation to subsequent readmission frequency (%)			*χ*^2^	*p*
	No readmission	1 readmission	≥ 2 readmissions		
Sepsis	3.4	5.1	6.5	47.1	< 0.001
Viral infections	0.5	0.3	0.2	3.3	0.194
Haematological disorders	2.2	1.1	1.0	20.3	< 0.001
Metabolic and endocrine disorders	3.3	4.0	4.3	6.5	0.039
Diabetes	1.0	1.6	2.2	20.8	< 0.001
Psychiatric disorders	0.8	1.2	2.1	23.8	< 0.001
Neurological disorders	2.3	1.8	2.7	3.2	0.198
Ophthalmic disorders	0.5	0.2	0.3	8.7	0.013
Cardiovascular disorders	13.7	13.3	13.0	0.9	0.630
Congestive heart failure^a^	1.1	2.6	3.2	80.1	< 0.001
Pulmonary disorders	9.4	16.8	21.3	279.3	< 0.001
Asthma^a^	0.7	0.5	0.5	1.6	0.439
Chronic obstructive pulmonary disease^a^	1.3	2.3	4.8	106.2	< 0.001
Pneumonia^a^	4.6	9.7	10.8	193.1	< 0.001
Gastrointestinal disorders	12.9	12.5	11.1	3.6	0.169
Dermatological disorders	3.4	2.9	3.4	1.7	0.436
Musculoskeletal disorders	10.5	5.1	2.7	144.4	< 0.001
Urological disorders	11.3	12.3	12.1	2.8	0.237
Urinary tract infection^a^	3.1	5.7	6.9	86.1	< 0.001
Bodily pain	13.8	11.9	8.4	31.0	< 0.001
Bone fractures	8.2	7.7	6.6	4.1	0.128
Hip fractures^a^	1.9	2.7	2.0	7.0	0.030
Medical device-related complications	3.1	3.7	4.3	8.0	0.019
Prolonged LOS in hospital (≥ 2 weeks)	7.8	21.6	39.3	1557	< 0.001

*LOS* length of stay

^a^Specific conditions within categories of disorders

### Association of readmission frequency on mortality

**Fig. 1:**
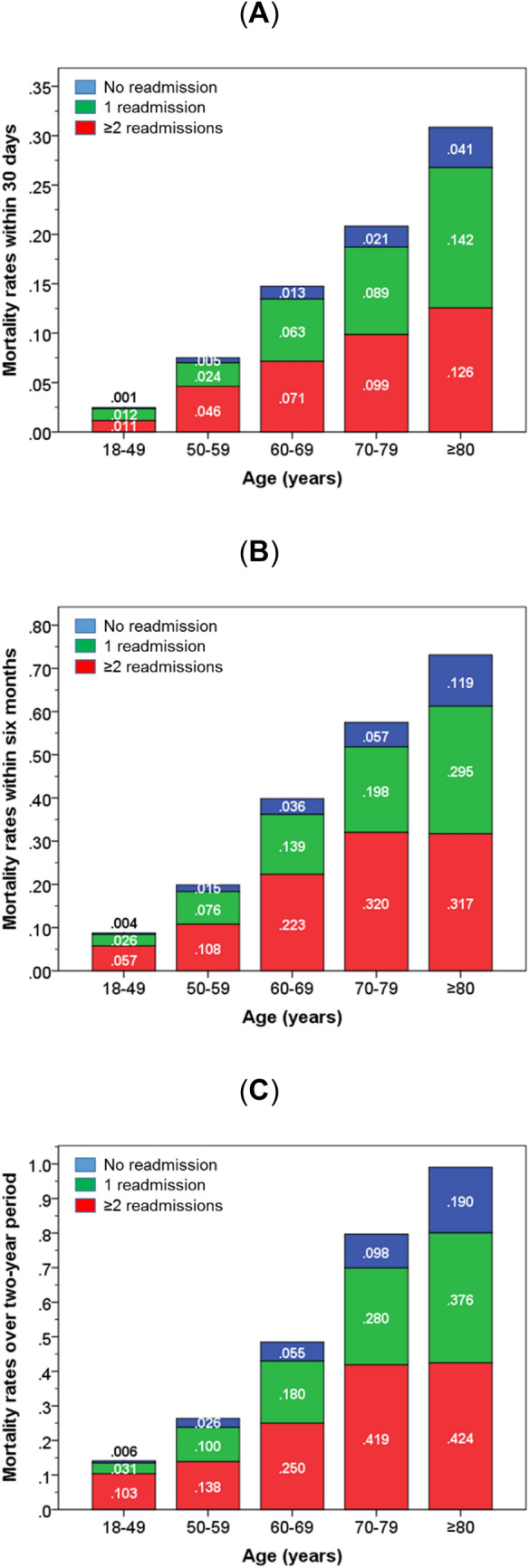
Stacked bar charts showing rates of mortality within 30 days (**a**) and 6 months (**b**) of discharge from first admission, and over a 2-year period (**c**) according to readmission status in different age categories. Note: the numbers represent the rates of mortality within each category of readmission status

Among those who were not readmitted, readmitted once, and readmitted ≥ 2 times within 28 days of discharge, the mortality rates within 30 days of discharge were 1.7, 9.2 and 10.0% (*χ*^2^ = 779, *p* < 0.001), and within 6 months of discharge were 4.8, 19.6 and 27.4% (*χ*^2^ = 1572, *p* < 0.001). Figure 1a–c shows that mortality rates increased progressively with age and increasing frequency of readmission. Within each age group of 18–49, 50–59, 60–69, 70–79 and ≥ 80 years, the mortality rates within 30 days of discharge for those who were not readmitted were 0.1, 0.5, 1.3, 2.1 and 4.1%; readmitted once were 1.2, 2.4, 6.3, 8.9 and 14.2%; and readmitted ≥ 2 times were 1.1, 4.6, 7.1, 9.9 and 12.6% (Fig. 1a). The corresponding mortality rates for those who were readmitted within 6 months of first discharge were: 0.4, 1.5, 3.6, 5.7 and 11.9% for no readmission; 2.6, 7.6, 13.9, 19.8 and 29.5% for one readmission; and 5.7, 10.8, 22.3, 32.0 and 31.7% (Fig. 1b). Within 2 years of discharge, these were 0.6, 2.6, 5.5, 9.8 and 19.0% for no readmission, 3.1, 1.0, 18.0, 28.0 and 37.6% for one readmission, and 10.3, 13.8, 25.0, 41.9 and 42.4% (Fig. 1c).

**Fig. 2:**
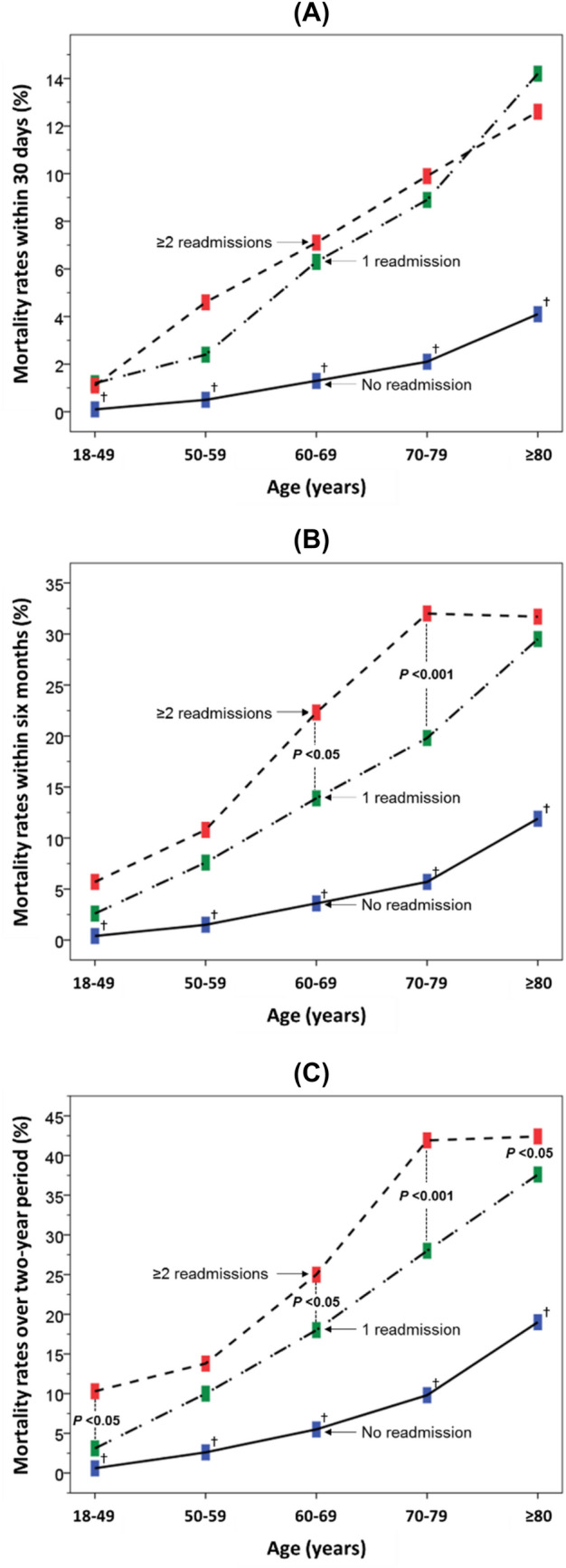
Mortality rates within 30 days (**a**) and 6 months (**b**) of discharge from first admission, and over a 2-year period (**c**) according to admission frequency in different age categories. Denotes differences from “no readmission group”: *p* < 0.001

The mortality rates within 30 days and 6 months of discharge, and over 2-year period all increased progressively with age, with higher rates for longer-term mortality (Fig. 2a–c). Analysis of short-term (within 30 days) mortality showed that compared with those who were not readmitted, patients who were readmitted just once or ≥ 2 times had similarly higher rates of mortality (Fig. 2a). When the analysis was extended to medium term (6 months) or long term (2-year period), mortality rates in those admitted once and ≥ 2 times uncoupled from each other with the widest gap among those aged 60–79 years (Fig. 2b, c).

Compared with patients with no readmission, the OR of mortality within 30 days of discharge in those who were readmitted once was 3.4 (95%CI = 2.9–4.0) and ≥ 2 times was 2.6 (95%CI = 2.0–3.3) for all ages, and for each age category from 18–49, 50–59, 60–69, 70–79 and ≥ 80 year: ORs were 10.7, 3.9, 4.0, 3.2 and 3.2 for 1 readmission and 10.2, 5.6, 3.7, 2.8 and 2.4 for ≥ 2 readmissions, respectively. The OR of mortality within 6 months of discharge in those who were readmitted once was 3.0 (95%CI = 2.7–3.4) and ≥ 2 times was 3.4 (95%CI = 2.9–4.0) for all ages, and for each age category from 18–49, 50–59, 60–69, 70–79 and ≥ 80 year: ORs were 7.5, 4.5, 3.3, 3.2 and 2.7 for 1 readmission and 12.4, 5.9, 4.1, 5.1 and 2.7 for ≥ 2 readmissions, respectively (Table 3).

**Table 3:** Stepwise multivariable logistic and Cox regression examining the association of readmission frequency and mortality, adjusted for age, sex and conditions presented as primary diagnosis (reported in Table 1) and prolonged LOS

Logistic regression	Age and sex adjusted mortality^a^							
	1 readmission^b^				≥ 2 readmissions^b^			
	Event rate (%)	OR	95% CI	*p*	Event rate (%)	OR	95% CI	*p*
Death within 30 days								
All ages	9.2	3.4	2.9–4.0	< 0.001	10.0	2.6	2.0–3.3	< 0.001
18–49 years	1.2	10.7	3.6–32.0	< 0.001	1.1	10.2	1.3–81.4	0.028
50–59 years	2.4	3.9	1.5–10.0	0.006	4.6	5.6	1.5–20.9	0.010
60–69 years	6.3	4.0	2.3–6.9	< 0.001	7.1	3.7	1.6–8.2	0.002
70–79 years	8.9	3.2	2.2–4.7	< 0.001	9.9	2.8	1.7–4.8	< 0.001
≥ 80 years	14.2	3.2	2.6–4.0	< 0.001	12.6	2.4	1.8–3.1	< 0.001
Death within 6 months								
All ages	19.6	3.0	2.7–3.4	< 0.001	27.4	3.4	2.9–4.0	< 0.001
18–49 years	2.6	7.5	3.4–15.4	< 0.001	5.7	12.4	4.3–35.5	< 0.001
50–59 years	7.6	4.5	2.6–7.8	< 0.001	10.8	5.9	2.5–14.2	< 0.001
60–69 years	13.9	3.3	2.3–4.8	< 0.001	22.3	4.1	2.5–6.9	< 0.001
70–79 years	19.8	3.2	2.5–4.2	< 0.001	32.0	5.1	3.6–7.1	< 0.001
≥ 80 years	29.5	2.7	2.2–3.2	< 0.001	31.7	2.7	2.2–3.3	< 0.001

Cox regression	Age and sex adjusted mortality^a^							
	1 readmission^b^				≥ 2 readmissions^b^			
	Event rate (%)	HR	95% CI	*p*	Event rate (%)	HR	95% CI	*p*
Death over two years								
All ages	25.5	2.2	2.0–2.4	< 0.001	36.1	2.5	2.2–2.8	< 0.001
18–49 years	3.1	4.9	2.6–9.0	< 0.001	10.3	12.4	5.8–26.6	< 0.001
50–59 years	10.0	3.3	2.1–5.1	< 0.001	13.8	3.7	1.8–7.5	< 0.001
60–69 years	18.0	2.7	2.0–3.6	< 0.001	25.0	2.9	1.9–4.4	< 0.001
70–79 years	28.0	2.5	2.0–3.0	< 0.001	41.9	3.5	2.7–4.4	< 0.001
≥ 80 years	37.6	2.0	1.8–2.3	< 0.001	42.4	2.2	1.9–2.5	< 0.001

^a^Age and sex adjustment for all analyses including individual age bands

^**b**^Reference group: No readmission; *OR* odds ratio; *HR* hazard ratio

**Fig. 3:**
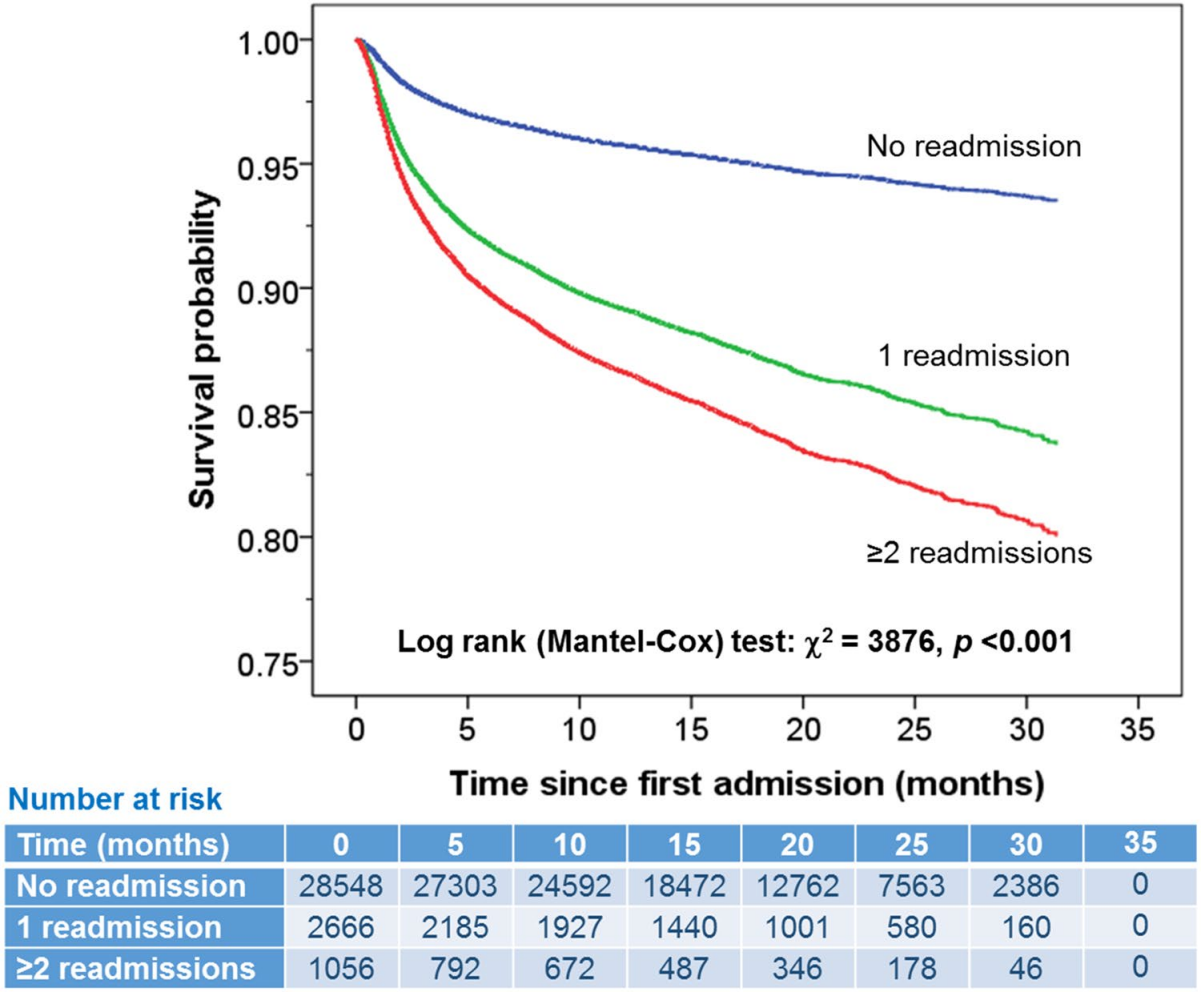
Kaplan–Meier survival plot in patients with different frequency of readmissions. The table beneath the figure shows the number of at-risk patients at various time points for the three readmission frequency cohorts

Compared to those with no readmission (mean for survival time from discharge = 30.2 months, 95%CI = 30.1–30.3), those who were readmitted once or ≥ 2 times had a significantly shorter survival with mean for survival time from discharge = 24.9 days (95%CI = 24.4–25.4) and 21.9 (95%CI = 21.0–22.7) days, respectively, log-rank (Mantel–Cox) test: *χ*^2^ = 3876, *p* < 0.001 (Fig. 3). Mortality over the 2-year period was increased for those who were readmitted once: event rate = 25.5%, HR = 2.2 (95%CI = 2.0–2.4), and ≥ 2 times: event rate = 36.1%, HR = 2.5 (95%CI = 2.2–2.8) for all ages, and for each age category from 18–49, 50–59, 60–69, 70–79 and ≥ 80 year: HRs were 4.9, 3.3, 2.7, 2.5 and 2.0 for 1 readmission and 12.4, 3.7, 2.9, 3.5 and 2.2 for ≥ 2 readmissions, respectively (Table 3).

## Discussion

This study found early emergency readmission frequency was a significant predictor of all-cause mortality within 30 days or 6 months post hospital discharge, and also over a 2-year period, in all age groups spanning 18–107 years, independent of presenting diagnosis, LOS, age and sex. Our findings are highly relevant both to clinical research and healthcare planning to further examine underlying reasons for the rising rates of early readmissions in England [5, 6].

We observed that short-term mortality rates (within 30 days of discharge) were similar for patients who were readmitted either once or for ≥ 2 times. However, mortality rates in those who were readmitted ≥ 2 times rose to a higher level when longer-term (within 6 months or over a 2-year period) mortality rates were examined. These differences could be explained, in part, by the higher prevalence of underlying conditions presented on first admission among those who were readmitted multiple times but who were able to survive over a short period, *i.e.* proportionally more of those readmitted ≥ 2 times die after 30 days of discharge than those who were readmitted once. Another explanation of this result could be that the 30-day mortality is an endpoint observed too early for patients readmitted ≥ 2 times, as these patients may have lived long enough to be admitted to the hospital two times or more. However, we did not have the timings between first hospital discharge and second readmission in this group of patients. It is likely that there are other reasons yet to be elucidated since readmission frequency predicts mortality independently of conditions presented on admission. This fundamental conundrum has been highlighted by Healthwatch England because the rates of readmissions continue to rise steadily in England without a clear explanation [5]. There are likely to be many factors contributing to this rise, including aspects related to patient discharge activities, such as the lack of explanation of the discharge plan provided to the patient, poor execution of discharge instructions, and lack of communication with primary care and coordination of care post-discharge, all of which are risk factors for avoidable readmissions [16]. Efforts have been made to reduce hospital readmissions, such as the Hospital Readmissions Reduction Program in the US, with mixed results [17]. A recent study showed that telephone contact with patients within 48 h of discharge lowered early readmission rates compared with those who had no contact (9.2% versus 15.7%, *p* = 0.011) [18]. Further research is therefore needed, focussing on safe discharge [19] and follow-up care for patients [20–22] and effective communication with primary care physicians [23]. The uncoupling of mortality rates between frequency of readmissions narrowed in the oldest age group may be due to higher rates of mortality or palliation in the community among this group which prevent them from readmissions.

Readmission frequency is routinely documented and assessed by secondary care centres as a way to identify frequent hospital users to provide care-support for patients in the community, [2] which both benefits patients and healthcare services. Evidence from this study further extends its utility as a simple and practical indicator of post-discharge mortality, even just a single readmission within 28 days of hospital discharge can identify those at high risk of death. The overall rates of readmission of 11.6% (8.3% once and 3.3% ≥ 2 times) observed in our study are at the lower end of those reported previously (11.6–18.4%) [24–26]. One factor is likely to be due to age differences between various study populations. As observed in our study of the incremental increase in readmission rates with age. The mortality rates observed in our study were also comparable to those recently reported for 30 days [27] and 6 months of discharge [28, 29]. As expected, the rates of mortality also increased with age. However, within each age category, the rates of mortality were consistently higher among individuals who were readmitted to hospital more frequently, suggesting that the risk of death from readmissions extends to the less-studied younger adults. Our recent study found that a risk of prolonged LOS in hospital (> 17 days) for patients admitted with hip fractures was increased by fourfold in those who acquired nosocomial pneumonia or a urinary tract infection [30]. This raises the possibility that patients who face emergency readmissions spend overall a much longer time in the risky hospital environment, such that mortality could, at least in part, be linked to hospital acquired complications. Conditions presented on admission may also play a key role in future readmissions and mortality. In the UK, the rates of admission with non-specific chest pain (22%) and abdominal pains (20%), urinary tract infection (11%), acute mental crisis (10%), COPD (10%) and angina (6%) are among the highest in patients > 75 years [31]. Excess admission rates have been reported to occur for pneumonia, congestive heart failure and COPD in older individuals and during the months of the influenza season [32].

The strengths of this study lie in its large number of consecutive patients, which enable us to estimate the risk of mortality by decades of age, ranging from 18 to 107 years. Appropriate adjustments were made including age, sex and presenting diagnoses. Characteristics of this study have been shown to be similar to those of the UK population [12, 33, 34]. There are certain limitations in the present study that may arise from patients who have left our catchment area or those who were readmitted to other centres this would lead to an underestimation of readmission rates. Studies have variably used between 28 and 30 days to define the period of early emergency readmission, [35] while we used 28 days for our definition, as guided by the NHS, [36] which would capture slightly lower numbers of readmissions. However, these differences do not affect the outcome of our studies since the primary purpose of our study was not intended for comparison of the performance of the frequency of readmissions with other studies.

In conclusion, early hospital readmission predicts short-, medium- and long-term mortality post-discharge from hospital in adults aged 18–107 years, independent of underlying presenting conditions, LOS, age and sex. Further research focussing on safe discharge and follow-up patient care may help reduce preventable or avoidable readmissions and post-discharge mortality.

## Abbreviations

CI: Confidence interval
ICD: International classification of diseases
LOS: Length of stay
NHS: National Health Service
OR: Odds ratio
HR: Hazard ratio

## References

[1] Benbassat J, Taragin M (2000). Hospital readmissions as a measure of quality of health care, advantages and limitations. Arch Intern Med.

[2] Nolte E, Roland M, Guthrie S, Brereton L (2012). Preventing emergency readmissions to hospital: a scoping review. Rand Health Q.

[3] Mayr FB, Talisa VB, Balakumar V, Chang CC, Fine M, Yende S (2017). Proportion and cost of unplanned 30-day readmissions after sepsis compared with other medical conditions. JAMA.

[4] Shah RM, Zhang Q, Chatterjee S, Cheema F, Loor G, Lemaire SA (2019). Incidence, cost, and risk factors for readmission after coronary artery bypass grafting. Ann Thorac Surg.

[5] Healthwatch (2018) Emergency readmissions: what's changed one year on? https://www.healthwatch.co.uk/report/2018-11-14/emergency-readmissions-whats-changed-one-year. Accessed 15 Nov 2020

[6] Healthwatch (2019) New plans to investigate rising emergency readmissions to hospitals announced in response to concerns raised by patients. https://www.healthwatch.co.uk/news/2019-01-17/new-plans-investigate-rising-emergency-readmissons-hospitals-announced-response. Accessed 15 Nov 2020

[7] Friebel R (2020) Trends in the number of English NHS hospital admissions, 2006 to 2016. The Health Foundation. https://www.health.org.uk/chart/chart-trends-in-the-number-of-english-nhs-hospital-admissions-2006-to-2016. Accessed 15 Nov 2020

[8] Karakusevic S (2016). Understanding patient flow in hospitals.

[9] Mudireddy P, Scott F, Feathers A, Lichtenstein GR (2017). Inflammatory bowel disease: predictors and causes of early and late hospital readmissions. Inflamm Bowel Dis.

[10] Agrawal S, Garg L, Shah M, Agarwal M, Patel B, Singh A (2018). Thirty-day readmissions after left ventricular assist device implantation in the United States: insights from the Nationwide Readmissions Database. Circ Heart Fail.

[11] Lewis KL, Fanaian M, Kotze B, Grenyer BF (2019). Mental health presentations to acute psychiatric services: 3-year study of prevalence and readmission risk for personality disorders compared with psychotic, affective, substance or other disorders. BJPsych Open.

[12] Fry CH, Heppleston E, Fluck D, Han TS (2020). Derivation of age-adjusted LACE index thresholds in the prediction of mortality and frequent hospital readmissions in adults. Intern Emerg Med.

[13] Heppleston E, Fry CH, Kelly K, Shepherd B, Wright R, Jones G (2020). LACE index predicts age-specific unplanned readmissions and mortality after hospital discharge. Aging Clin Exp Res.

[14] World Health Organization (2004). ICD-10: international statistical classification of diseases and related health problems: tenth revision.

[15] https://digital.nhs.uk/data-and-information/publications/clinical-indicators/nhs-outcomes-framework/archive/nhs-outcomes-framework-indicators---february-2019-release. Accessed 15 Nov 2020

[16] Aspenson M, Hazary S (2012). The clock is ticking on readmission penalties. Healthc Financ Manag.

[17] Wadhera RK, Maddox KE, Wasfy JH, Haneuse S, Shen C, Yeh RW (2018). Association of the Hospital Readmissions Reduction Program with mortality among Medicare beneficiaries hospitalized for heart failure, acute myocardial infarction, and pneumonia. JAMA.

[18] Vernon D, Brown JE, Griffiths E, Nevill AM, Pinkney M (2019). Reducing readmission rates through a discharge follow-up service. Future Healthc J.

[19] Wong SP, Sharda N, Zietlow KE, Heflin MT (2020). Planning for a safe discharge: more than a capacity evaluation. J Am Geriatr Soc.

[20] Agostinho JR, Gonçalves I, Rigueira J, Aguiar-Ricardo I, Nunes-Ferreira A, Santos R (2019). Protocol-based follow-up program for heart failure patients: Impact on prognosis and quality of life. Rev Port Cardiol.

[21] Catanach B, Betz ME, Tvrdy C, Skelding C, Brummett S, Allen MH (2019). Implementing an emergency department telephone follow-up program for suicidal patients, successes and challenges. Jt Comm J Qual Patient Saf.

[22] de Mestral C, Kayssi A, Al-Omran M, Salata K, Hussain MA, Roche-Nagle G (2019). Home care nursing after elective vascular surgery, an opportunity to reduce emergency department visits and hospital readmission. BMJ Qual Saf.

[23] Destino LA, Dixit A, Pantaleoni JL, Wood MS, Pageler NM, Kim J (2017). Improving communication with primary care physicians at the time of hospital discharge. Jt Comm J Qual Patient Saf.

[24] Gruneir A, Dhalla IA, van Walraven C, Fischer HD, Camacho X, Rochon PA (2011). Unplanned readmissions after hospital discharge among patients identified as being at high risk for readmission using a validated predictive algorithm. Open Med.

[25] Lim E, Matthew N, Mok W, Chowdhury S, Lee D (2011). Using hospital readmission rates to track the quality of care in public hospitals in Singapore. BMC Health Serv Res.

[26] Tan SY, Low LL, Yang Y, Lee KH (2013). Applicability of a previously validated readmission predictive index in medical patients in Singapore, a retrospective study. BMC Health Serv Res.

[27] Dharmarajan K, Wang Y, Lin Z, Normand SL, Ross JS, Horwitz LI (2017). Association of changing hospital readmission rates with mortality rates after hospital discharge. JAMA.

[28] Mazzola P, Bellelli G, Broggini V, Anzuini A, Corsi M, Berruti D (2015). Postoperative delirium and pre-fracture disability predict 6-month mortality among the oldest old hip fracture patients. Aging Clin Exp Res.

[29] Park LP, Chu VH, Peterson G, Skoutelis A, Lejko-Zupa T, Bouza E (2016). Validated risk score for predicting 6-month mortality in infective endocarditis. J Am Heart Assoc.

[30] Lisk R, Uddin M, Parbhoo A, Yeong K, Fluck D, Sharma P, Lean ME, Han TS (2019). Predictive model of length of stay in hospital among older patients. Aging Clin Exp Res.

[31] O'Cathain A, Knowles E, Maheswaran R, Pearson T, Turner J, Hirst E, Goodacre S, Nicholl J (2014). A system-wide approach to explaining variation in potentially avoidable emergency admissions: national ecological study. BMJ Qual Saf.

[32] Yap FH, Ho PL, Lam KF, Chan PK, Cheng YH, Peiris JS (2004). Excess hospital admissions for pneumonia, chronic obstructive pulmonary disease, and heart failure during influenza seasons in Hong Kong. J Med Virol.

[33] Han TS, Fry CH, Fluck D, Affley B, Gulli G, Barrett C (2017). Evaluation of anticoagulation status for atrial fibrillation on early ischaemic stroke outcomes, a registry-based, prospective cohort study of acute stroke care in Surrey, UK. BMJ Open.

[34] Han TS, Fry CH, Gulli G, Affley B, Robin J, Irvin-Sellers M, Fluck D (2020). Prestroke disability predicts adverse poststroke outcome: a registry-based prospective cohort study of acute stroke. Stroke.

[35] Zhou H, Della PR, Roberts P, Goh L, Dhaliwal SS (2016). Utility of models to predict 28-day or 30-day unplanned hospital readmissions: an updated systematic review. BMJ Open.

[36] NHS Digital (2010) Archived Emergency readmissions to hospital within 28 days of discharge, financial year 2010/11. https://files.digital.nhs.uk/BB/42FA85/hes-emer-read-hosp-28-days-disc-2001-2011-pra.pdf. Accessed 15 Nov 2020

